# Bayesian Estimation of Oscillator Parameters: Toward Anomaly Detection and Cyber-Physical System Security

**DOI:** 10.3390/s22166112

**Published:** 2022-08-16

**Authors:** Joseph M. Lukens, Ali Passian, Srikanth Yoginath, Kody J. H. Law, Joel A. Dawson

**Affiliations:** 1 Quantum Information Science Section, Oak Ridge National Laboratory, Oak Ridge, TN 37831, USA; 2 Systems and Decision Sciences Group, Oak Ridge National Laboratory, Oak Ridge, TN 37831, USA; 3 Department of Mathematics, University of Manchester, Manchester M13 9PL, UK; 4 Energy and Control Systems Security Group, Oak Ridge National Laboratory, Oak Ridge, TN 37831, USA

**Keywords:** Bayesian estimation, cyber-physical security, dynamical systems, sensors and actuators

## Abstract

Cyber-physical system security presents unique challenges to conventional measurement science and technology. Anomaly detection in software-assisted physical systems, such as those employed in additive manufacturing or in DNA synthesis, is often hampered by the limited available parameter space of the underlying mechanism that is transducing the anomaly. As a result, the formulation of anomaly detection for such systems often leads to inverse or ill-posed problems, requiring statistical treatments. Here, we present Bayesian inference of unknown parameters associated with a generic actuator considered as a representative vital element of a cyber-physical system. Via a series of experimental input-output measurements, a transfer function for the actuator is obtained numerically, which serves as our model for the proposed method. Linear, nonlinear, and delayed dynamics may be assumed for the actuator response. By devising a code-based malicious signal, we study the efficacy of Bayesian inference for its potential to produce a detection, including uncertainty quantification, with a remarkably small number of input data points. Our approach should be adaptable to a variety of real-time cyber-physical anomaly detection scenarios.

## 1. Introduction

Complex systems are known to pose significant difficulties to analytical modeling and analysis. The multiple couplings and parameter dependencies drive the challenges even into the computational domain, where coupling parameters are either unknown or lack sufficient quantitative representations. These challenges are exacerbated when extending the modeling considerations into a security regime where one attempts to predict, identify, or prevent any deviations in the operation of the complex systems and networks. Sensors and actuators, comprising key components of many scientific and technological systems, are increasingly integrated with software and cyber systems to form complex systems [1]. The physics of sensors actively produce new concepts and solutions commensurate with the evolving needs for in vivo, in vitro, in situ, and environmental measurements. Furthermore, with emerging trends in metrology and artificial intelligence, and associated applications in quantum sensing and edge computing [1], the horizon is teeming with countless powerful interactive sensors and actuators. Consequently, cyber-physical system (CPS) security for device protection and quality control is urgently needed across many industrial and infrastructural systems.

The physical systems for which detection of malicious activities are needed are diverse. For example, as energy consumption increases across the globe, effective exploitation of transactive energy [2], that is, the peer-to-peer sharing and trading of energy, requires safeguarding. Zhang et al. [3] recently developed cyber-attack models for transactive energy, where detection of anomalies in the market and physical system measurements (e.g., voltage and frequency or other operational parameters of the system) are sought. Given the rapidly growing number of sensors and other subsystems (e.g., the components associated with edge computing [1]), one may capitalize upon established physical theories known for their ability to handle statistically large numbers of subsystems. In one such approach, Tavolato et al. [4] employed kinetic gas theory to model anomaly detection in networks, with which the system response may be investigated as a whole, rather than at the level of individual subsystems. By modeling the system as a multi-agent network, Tavolato et al. predicted and measured the CPS operation attributes and determined how they deviate from each other. The deviation provides a basis to issue an alert regarding a potential attack or malfunction. Other examples include context-sensitive modeling by Saez et al. [5], in which physics-based and data-driven models were investigated for anomaly detection for the given hardware or process. Such physics-based approaches require setting up dynamic equations for the involved machines, data extraction, and signal processing.

Examples of important CPS classes include synthetic biology [6] and biotechnological instruments and devices operating bioinformatics programs, such as DNA synthesizers. A DNA synthesizer enables custom-building of sequences of oligonucleotides or short DNA strands using the A, G, C, and T nucleobases. Recognizing the cybersecurity vulnerabilities of DNA synthesis, recent investigations have addressed attack feasibility, as demonstrated by Faezi et al. [7], who explored an acoustic side-channel attack methodology on DNA synthesizers. Earlier studies by Nei et al. [8], who reported a security analysis of the DNA processing pipeline, demonstrated DNA-based exploits as well. Exploring measurement science and technology to devise novel experimental detection schemes is gaining prominence, as noted in recent works by Gatlin et al. [9] and Yarnpolskiy et al. [10]. By monitoring the electric currents supplied to actuators (printer motors) employed in a manufacturing process, Gatlin et al. described anomaly detection by comparison to current consumption during normal processes. Similar considerations have been reported by Ranabhat et al. [11], with a focus on composite materials (e.g., carbon fiber-reinforced polymer) being used as functional parts in safety-critical systems.

Here, we propose a Bayesian analysis of the response of a simple exemplar CPS. The response is here taken to be in the form of time-series data acquired, for simplicity, from voltage measurements in typical electro-mechanical hardware components. To motivate further work toward anomaly detection for CPS security, we estimate the frequency content of a potentially malicious input. Our approach applies the inherent advantages of Bayesian spectrum estimation to the specific use case of CPS, complementing previous Bayesian research and application domains drawing on belief networks and game theory [12,13,14].

The article is organized as follows. In Section 2, we review some relevant definitions and introduce an example generic CPS problem to be tackled with Bayesian inference. Here, for the sake of presentation, we first introduce the CPS as a simple actuator modeled as a damped driven harmonic oscillator, defining the difference between normal and anomalous operation for our purposes. We then overview the Bayesian inference method, including details of the MCMC sampling procedure that enables efficient numerical evaluation. In Section 3, we introduce a real-life actuator as a case study; by measuring the input-output signals from it, we obtain a system transfer function to be used as the model in the Bayesian test. We then performed Bayesian inference on realistic output data using the obtained transfer function. By estimating the ground truth frequency, we found exceptional accuracy with our technique: sub-Hz discrimination with only around 5 ms of time samples. This situation was made possible by the power of Bayesian spectrum estimation, which is able to sift signals amid noise through a logical framework absent in typical fast Fourier transform (FFT) approaches. This section ends by exploring the envisioned scenario in which the Bayesian results are summarized into a single number—the anomaly probability—which is provided to the user for decision making. Section 4 concludes the paper.

## 2. Problem Formulation

Cyber-attacks, traditionally targeting information technology systems, lead to theft and tampering of non-physical digital entities, such as data and intellectual property. A CPS constitutes an arrangement of cyber (e.g., data and algorithms) and physical components (e.g., actuators and machine parts). Thus, unlike traditional cyber-attacks, attacks targeting CPS also compromise a *physical* system. A CPS can be an elaborate mixture of many components, the formulation of which represents a complex problem requiring knowledge about the underlying statics and dynamics, often leading to coupled nonlinear systems of partial differential equations, representing a multi-physics problem. This problem becomes quickly more complicated by incorporating stochastic and memory effects (e.g., noise and delayed feedback), requiring stochastic delay differential equations [15]. Integration of sensor dynamics, data, and system information and knowledge can aid in devising new anomaly detection approaches. An example of a specialized but important CPS is a 3D printer employed in additive manufacturing. Here, we begin by discussing a more generic CPS, simply modeled as a noisy and driven linear second-order system. Clearly, this is a highly simplified case study, but it allows us to explore the complexity of the resulting problem so that generalization toward more practically relevant problems can be made.

### 2.1. SHO Actuator

To set up our basic analysis use-case, we first note that many components of machines and industrial systems are designated to perform some form of harmonic or anharmonic movement. The resulting actuation is often measured using sensors, the output data of which may be used to impose constraints or control on the actuators. A CPS may therefore include smart networked subsystems with embedded sensors, processors, and actuators that sense and interact with the physical world in real-time. Naturally, the mathematical description of the resulting dynamics can quickly become complicated, requiring many interrelated and coupled partial differential equations (PDEs). Without a model-based, analytical, numerical, or computational solution, devising security measures against malfunction will be challenging. Quite often, the dynamics of complicated systems may be described by invoking simpler subsystems, which in many cases can be approximated by harmonic motion so that the equation of a simple harmonic oscillator (SHO) can be used—a second-order ordinary differential equation (ODE). Often, some aspects of more complicated PDEs can be reduced ODEs as well. Therefore, an SHO forms a natural first step to studying more complicated dynamical systems, such as a CPS. In the simpler discrete form, an SHO describes the motion of a particle of mass *m* at a given position *y* and time *t*. When a force is applied to the particle, it responds elastically according to Hooke’s law, perhaps accompanied by a damping mechanism proportional to the particle velocity.

### 2.2. A Simplified CPS Model

**Figure 1:**
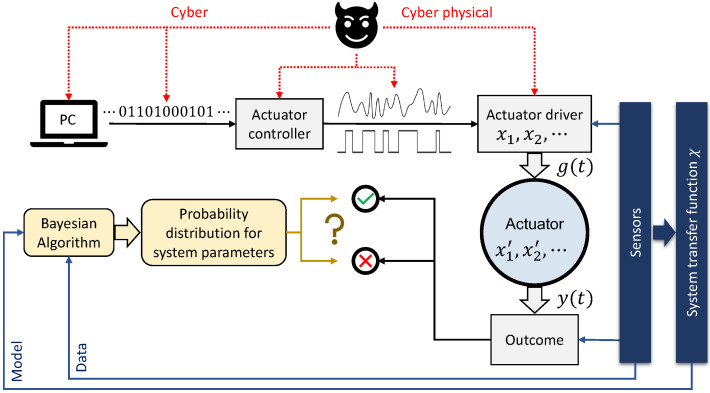
Schematic of the proposed Bayesian anomaly detection approach. A set of digital instructions may be converted to an analog input g(t) driving a linear or nonlinear actuator. Parameters $x_{i}^{\prime }$ ($x_{i}$), i=1,2,… describe the actuator (actuator input). An array of sensors measure the input-output relation and generate a transfer function χ, which is utilized as a model by the Bayesian algorithm. The model and the outcome data y(t) are employed by the Bayesian algorithm to generate probability distributions for the parameters involved. Such an analysis has the potential to detect an adversarial influence on the outcome from either a cyber or a cyber-physical attack (an example being a modification of the G-code in 3D printing [18]).

The proposed anomaly detection approach requires both measurement data and a system model. The overall CPS configuration is shown in Figure 1, where an actuator generically shows the physical system, intended to guide the description of our approach. We assume our system to be composed of a single damped harmonic oscillator of mass *m*, damping γ, and stiffness *k*. The actuator is driven by a time *t* dependent force g(t), which is composed of a deterministic and a stochastic part, f(t) and ξ(t), respectively, with the latter representing effects on the actuator that can only be described probabilistically. We assume the actuator state y=y(t) obeys the Langevin equation:

(1) $$L_{0}y\left(t\right)=g\left(t\right)=b_{0}\xi \left(t\right)+b_{1}f\left(t\right),$$

where $L_{0}=\partial_{t}^{2}+2\Gamma \partial_{t}+\omega_{0}^{2}$ is the harmonic oscillator differential operator; Γ=γ/(2m), $\omega_{0}=\sqrt{k/m}$, and $b_{0}$ and $b_{1}$ are constants. The function ξ represents thermal white noise so that:

(2) $$\langle \xi \left(t\right)\rangle =0,\;\;\;\mathrm{and}\;\;\;\;\;\langle \xi \left(t\right)\xi \left(t^{\prime }\right)\rangle =\delta \left(t-t^{\prime }\right),$$

i.e., zero mean and delta-function correlated. Denoting the frequency by ω, and taking the Fourier transform of Equation (1), gives:

(3) $$Y\left(\omega \right)=\chi \left(\omega \right)G\left(\omega \right)=\frac{F\{b_{0}\xi \left(t\right)+b_{1}f\left(t\right)\}\left(\omega \right)}{\omega_{0}^{2}-2i\Gamma \omega -\omega^{2}},$$

where χ(ω) is the complex susceptibility (transfer function) describing the frequency response of the system, and G(ω) describes the driving force in the Fourier domain. Then for f(t)=cosωt, and employing the fluctuation-dissipation theorem (setting $mb_{0}=\sqrt{2\gamma K_{B}T}$, where $K_{B}$ is the Boltzmann constant and *T* is the temperature) [16], the stationary state of the actuator can be shown to be given by [17]:

(4) $$\langle y^{2}\left(t\right)\rangle =\frac{K_{B}T}{k}+b_{1}^{2}{\left[\frac{2\Gamma \omega \sin\left(\omega t\right)+\left(\omega_{0}^{2}-\omega^{2}\right)\cos\left(\omega t\right)}{{\left(\omega^{2}-\omega_{0}^{2}\right)}^{2}+4\Gamma^{2}\omega^{2}}\right]}^{2}.$$

If one assumes that the system is in a stationary state, then a sudden tampering may lead to a transient response, potentially followed by resumption of a stationary state. Since application of the fluctuation-dissipation theorem assumes that the system is in equilibrium, any out-of-equilibrium state leads to a deviation from the closed-form expression for the noise.

The above considerations may be extended to nonlinear systems, although obtaining a response function can be significantly more challenging. A promising nonlinear oscillator model for the description of many actuators is the driven noisy Duffing equation, which is obtained by modifying $L_{0}\to L_{0}+ay^{2}\left(t\right)$, where *a* sets the strength of the nonlinearity. In principle, one may link an algorithm for solving the differential equation as a "model" for the Bayesian analysis. For example, the Duffing equation above may be solved numerically to study the oscillator phase diagram [19] or the stochastic resonance [20]. In such cases where the forward model is not in closed form—i.e., an explicit likelihood expression such as Equation (7) below is not available—multilevel Monte Carlo techniques and their extensions seem particularly promising to pursue [21,22]. Other scenarios amenable to treatment by the Bayesian method and of interest to actuator dynamics include systems where delays (pure time delays, constant delays, phase shifts, etc.) cannot be neglected. Whether through feedback with gain and delay, or through a delay coupling, the eigenfrequency spectrum of the actuator will be affected. Nonlinear systems, including those due to delays, will be the subject of future work.

### 2.3. Normal Versus Anomalous System Operation and Anomaly Detection

For the sake of presentation, we define an anomaly as follows. With reference to Figure 1, a deviation from established or desired parameter ranges, either for those describing the input signal (x) or for those describing the actuator itself ($x^{\prime }$), constitutes an anomaly or an outlier event. Here, one may consider any plausible entry point for a source of undesired operation or action that may affect a system parameter unfavorably. For a single parameter, if the new value is outside an agreed-upon normal range, then a flag is raised. For multiple altered parameters within nominal ranges, one may seek to analyze other health assessors that may be sensitive to a bad combination of altered within-nominal-range parameters. This approach is similar to the established threat or fault modeling techniques in cybersecurity and process engineering [23,24].

An enormous body of work has been performed on statistical techniques and artificial intelligence toward detection of system behavior, including those caused by malicious sources. We now proceed to apply Bayesian reasoning to this significant problem.

### 2.4. Bayesian Model

We assume that a sensor measures and digitizes some output voltage y(t) at a fixed time interval Δt, so that a collection of *N* such data samples $y=(y_{0},y_{1},\ldots ,y_{N-1})$ corresponds to observations at times $t_{n}=n\Delta t$ (n∈{0,1,…,N−1}). From these samples, the goal is to estimate the underlying properties of the system and return the probability that they deviate from an acceptable range of operation, thereby indicating tampering or failure.

The Bayesian formalism offers a principled path to a unique answer for such a well-posed problem [25]. In our case, we assume an attack surface covering the input signal to the actuator (parameters x in Figure 1), but take the actuator itself as characterized and secure; we make this assumption for clarity in the proof-of-principle examples here, but the approach can readily be extended to arbitrary system parameters. Then, the probability density π(x) given the *N* datapoints in y follows Bayes’ theorem as

(5) $$\pi \left(x\right)=\frac{1}{Z}L_{y}\left(x\right)\pi_{0}\left(x\right)$$

where the likelihood $L_{y}\left(x\right)\propto \mathrm{Pr}\left(y|x\right)$ (the probability of observing data y given parameters x); $\pi_{0}\left(x\right)$ is the prior distribution, which describes allowed values of x assumed before data collection; and Z is a normalizing constant to ensure ∫dxπ(x)=1, which need not be computed in the numerical techniques below. In order to obtain an expression for $L_{y}\left(x\right)$, we first write the output waveform as

(6) y(t)=Acos(ωt+α)+ϵ(t),

with a noise term ϵ(t). This formula assumes (i) a single-frequency input signal, which is carried to the output if the system is linear, and (ii) additive noise similar to that introduced in Section 2.2. If we make the typical and conservative [26] assumption of white Gaussian noise with variance $\sigma^{2}$, then the samples $y_{n}=y\left(t_{n}\right)$ are independent, and the likelihood follows as

(7) $$L_{y}\left(x\right)=\frac{1}{\sigma^{N}}\prod_{n=0}^{N-1}\exp\left\{-\frac{{\left[y_{n}-A\cos\left(\omega t_{n}+\alpha \right)\right]}^{2}}{2\sigma^{2}}\right\},$$

enumerating all unknown parameters through x=(ω,A,α,σ). Finally, in order to impose a minimal amount of prior knowledge, we assume that any value within a predefined range for each parameter is equally probable, i.e.,

(8) $$\pi_{0}\left(x\right)\propto {𝟙}_{\left(0,\omega_{M}\right)}\left(\omega \right){𝟙}_{\left(0,A_{M}\right)}\left(A\right){𝟙}_{\left(-\pi ,\pi \right)}\left(\alpha \right){𝟙}_{\left(0,\sigma_{M}\right)}\left(\sigma \right),$$

where the indicator function ${𝟙}_{\left(a,b\right)}\left(x\right)$ equals one whenever *x* lies in the interval (a,b), and zero otherwise.

Now, the four-dimensional integration required to compute parameter estimates from the complete probability density π(x) [Equation (5)] cannot be performed analytically on this combination of likelihood and prior—a typical situation in Bayesian inference—so we invoke Markov chain Monte Carlo (MCMC) techniques [25,27] to numerically draw *R* samples $x^{\left(r\right)}$ from π(x). Then, the Bayesian expectation of any function of x can be estimated directly as

(9) $$\phi_{B}=\langle \phi (x)\rangle \approx \frac{1}{R}\sum_{r=1}^{R}\phi \left(x^{\left(r\right)}\right),$$

which is the optimal estimator in terms of attaining the minimum squared error with respect to the ground truth, when averaged over all parameter values and possible outcomes [27]. Indeed, in addition to automatic uncertainty quantification, this optimality represents one of the fundamental advantages of Bayesian methods in general and in practice can lead to massive improvements in accuracy over more conventional methods—a feature that will help explain some of the striking results in the spectrum estimation examples below.

As our specific MCMC procedure, we employed the preconditioned Crank–Nicolson (pCN) algorithm [28], a special case of Metropolis–Hastings [29,30] which mitigates the "curse of dimensionality"—the inherent acceptance rate reduction with dimension that faces random walk techniques. The details of our pCN algorithm are beyond the scope of the present study, but we point the reader to [31] for useful background on pCN in the context of quantum state tomography, and to [32], where we incorporate the Markov chain proposal of [33] to permit the use of pCN techniques on uniform priors, such as those in Equation (8). In fact, the MCMC procedure followed here is identical to that in [32], modified only with the different likelihood in Equation (7).

In the context of cyber-physical security, of critical importance is how our Bayesian inference procedure performs with the number of samples *N*. In order to detect the presence of an anomaly quickly and initiate appropriate countermeasures in real time, one would like to obtain low-uncertainty estimates with as few samples as possible. Ultimately, the number of samples required will depend on a variety of configuration- and application-specific characteristics, including the system noise level and the relative cost of either an undetected anomaly or a false alarm. Importantly, however, the mean-squared optimality of the Bayesian mean holds for *any* fixed *N*, a feature which supplies strong theoretical justifications for the estimator as a whole, while not obviating the need to address a variety of questions in a specific platform.

## 3. Proof-of-Principle Example

### 3.1. Experimental Test CPS

An example of an actual but simple actuator is an electro-mechanical rotator with a turning shaft that is controlled to elicit certain behavioral qualities, including angular speed, torque, and the direction and distance of rotation. We instantiated this in a small testbed that, consistently with Figure 1, translates discrete instructions into a series of pulse-width-modulated (PWM) signals, one for each phase of the motor. Each PWM switches a Darlington pair, which then closes a circuit and energizes the corresponding phase of the motor, in turn rotating the shaft.

To understand this system analytically, we applied a simple model for a representative DC armature motor. With the definitions given in Table 1, we can represent the motor’s primary action with the following Laplace *s*-domain equations:

(10) $$I_{a}\left(s\right)=\frac{E_{a}\left(s\right)-E_{b}\left(s\right)}{L_{a}s+R_{a}},$$

(11) $$T\left(s\right)=K_{T}I_{a}\left(s\right),$$

(12) $$\Omega_{m}\left(s\right)=\frac{T(s)}{J_{m}s+B_{m}},$$

which yield the motor’s steady state current $I_{a}\left(s\right)$, torque T(s), and shaft angular speed (in rad/s) $\Omega_{m}\left(s\right)$, respectively. These can be combined into the following second-order transfer function:

(13) $$\chi \left(s\right)=\frac{\Omega_{m}\left(s\right)}{E_{a}\left(s\right)}=\frac{A}{s^{2}+Bs+C},$$

where

(14) $$\begin{array}{ccc} A & = & \frac{K_{T}}{L_{a}J_{m}}, \end{array}$$

(15) $$\begin{array}{ccc} B & = & \frac{R_{a}J_{m}+N_{m}L_{a}}{L_{a}J_{m}}, \end{array}$$

(16) $$\begin{array}{ccc} C & = & \frac{K_{T}K_{E}+R_{a}B_{m}}{L_{a}J_{m}}. \end{array}$$

The subscripts *a* and *m* indicate, respectively, variables pertaining to the armature’s electrical dynamics and the motor’s mechanical dynamics.

**Figure 2:**
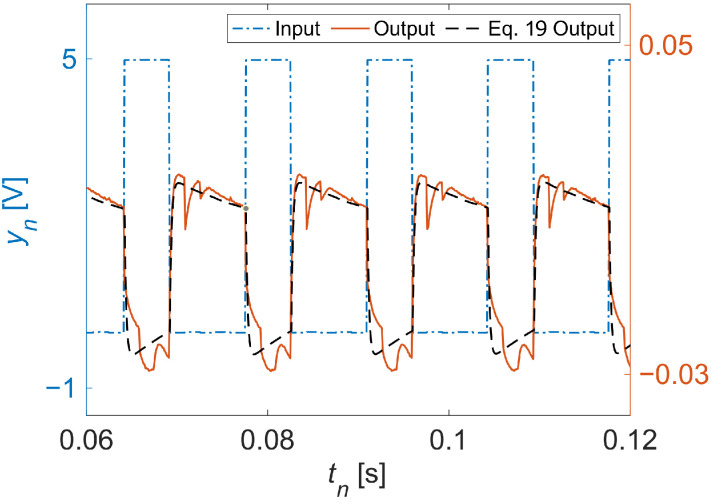
Dynamics of the studied actuator. Shown are the experimentally measured output signal, which is the actuator response to a periodic square-wave input signal, and the simulated output of Equation (19) given the same input signal.

**Figure 3:**
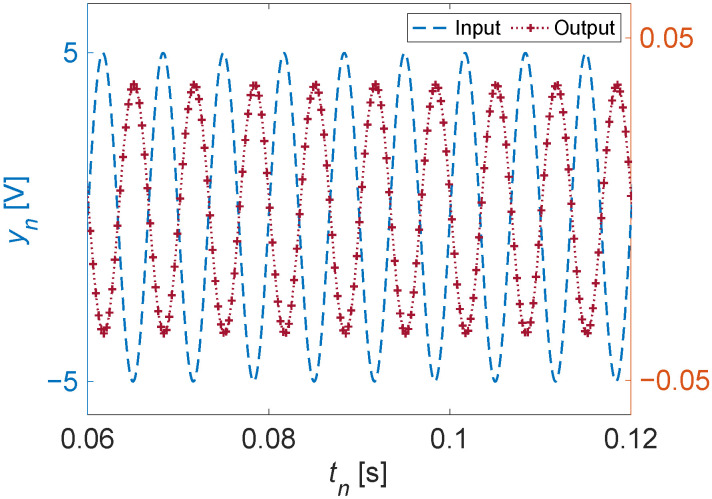
Estimated output data for the considered actuator, given an example input, using the generated transfer function [Equation (17)].

Alternatively, we may numerically determine the corresponding transfer function using measurement data acquired from our experiments. For our system’s input and output signals, we selected, respectively, the PWM control signal and coil current flow from a single phase of our testbed’s motor. We recovered both signals as voltage measurements due to the use of a passive current transducer on the coil lead. Both the measured input and output data are shown in Figure 2. Using this data, we could numerically obtain a transfer function with a polynomial optimization algorithm [34]. Given the theory behind Equation (13), we chose a generic second-order fit for χ(s) of the form:

(17) $$\chi \left(s\right)=\frac{as+b}{s^{2}+cs+d},$$

where (a,b,c,d)=(−39.65,17.82,5416,348600) (*b* and *d* are dimensionless; *a* and *c* are measured in seconds). The system’s response to an arbitrary input, such as a harmonic input of amplitude $A_{e}$, frequency $\omega_{e}$, and phase $\alpha_{e}$, expressed in the Laplace domain as

(18) $$G\left(s\right)=\left(\frac{\omega_{e}\cos\alpha_{e}+s\sin\alpha_{e}}{s^{2}+\omega_{e}^{2}}\right)A_{e},$$

may now be readily obtained from:

(19) $$Y\left(s\right)=\chi \left(s\right)G\left(s\right)=\frac{\left(\omega_{e}\cos\alpha_{e}+s\sin\alpha_{e}\right)\left(as+b\right)}{\left(s^{2}+\omega_{e}^{2}\right)\left(s^{2}+cs+d\right)}A_{e},$$

which when inverse-transformed, may be used to visualize the response, as shown in Figure 3 for a frequency of $\omega_{e}/2\pi =150$ Hz. It is worth noting that the variable *s* in the Laplace transform is in general complex: $s=s_{r}+i\omega$. When *s* is purely imaginary s=iω, then the Laplace transform reduces to the Fourier transform. In addition to analytical convenience, the choice of the transform can also be motivated by the existence of a transform for a given function in one domain versus the other. (In a slight abuse of notation, we use the same symbols for both Fourier and Laplace, letting the argument *s* or ω show which transform is implied.)

### 3.2. Bayesian Inference Results

**Figure 4:**
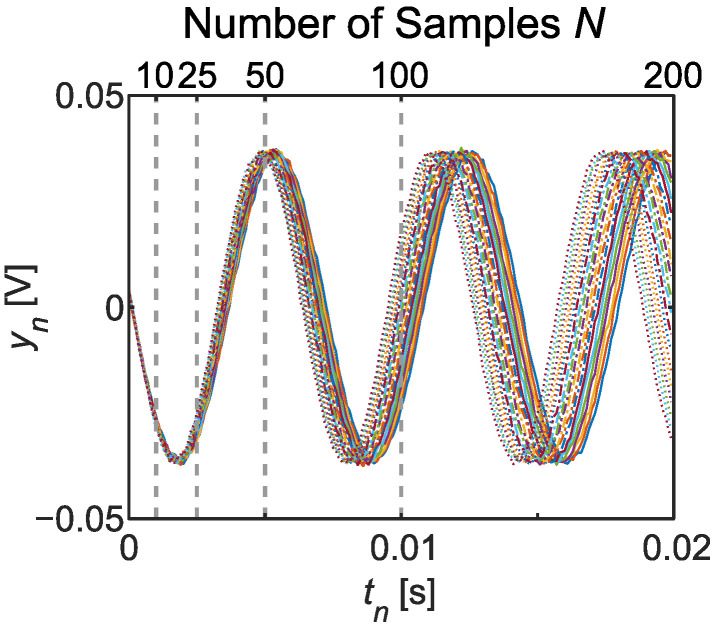
Output voltage samples simulated from a stepper motor excited by 21 sinewaves with frequencies evenly spaced from 140 to 160 Hz. Vertical dashed lines denote the total durations of subsets with various numbers of samples *N*. (See legend in Figure 5 for the ground truth frequency corresponding to each combination of color and line style.)

**Figure 5:**
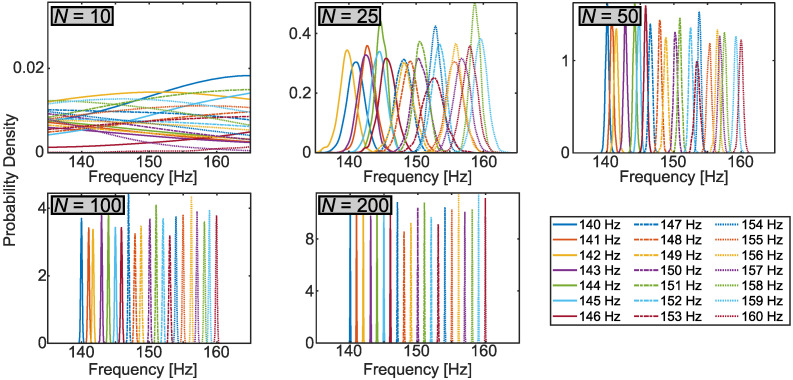
Marginal posterior distributions of excitation frequency, obtained by Bayesian inference of the datasets in Figure 4 and grouped by the number of data samples *N*. The ground truth frequencies for each curve appear in the legend.

We simulated the output for input sinewaves of varying frequencies from 140 to 160 Hz, with a sensor sampling rate of 10 kHz (Δt=100μs) and output noise standard deviation of $\sigma_{G}=0.5$ mV. Plots of all data vectors y appear in Figure 4. Each curve corresponds to a specific ground truth frequency $f_{G}=\omega_{G}/2\pi \in \left\{140,141,\ldots ,160\right\}$ Hz (see legend in Figure 5). Dotted vertical lines delineate the different sample sets considered for inference; e.g., N=10 signifies that the first 10 samples starting with $t_{0}=0$ s were included, and N=200 that all 200 samples from 0 to 20 ms comprise y. Thus, a total of 105 MCMC inference results were separately obtained, accounting for all five values of *N* and 21 frequencies.

For the prior, we took $\omega_{M}/2\pi =5$ kHz (the maximum non-aliased frequency for 10 kHz sampling by the Nyquist theorem) and $A_{M}=\sigma_{M}=100$ mV to fully encompass the voltage scale in our system. We kept $R=2^{10}$ samples from a total chain length of RT, where the thinning factor of $T=2^{19}$ was found sufficient empirically to ensure that all parameter means and variances had converged to final values. The sample sets allowed estimates of any of the four parameters (ω,A,α,σ); yet for the purposes of this test, we focused on frequency specifically. Taking the *R* samples $\left\{\omega^{\left(r\right)}\right\}$ obtained for each dataset, we computed an estimate of the marginal probability density for frequency using the built-in kernel smoothing function in MATLAB [35]. Figure 5 plots all 21 probability densities for each sample number *N* as a function of cyclic frequency f=ω/2π. While the N=10 case returned extremely broad distributions that were on top of each other (a consequence of insufficient data to identify frequency), clear peaks appeared for just N=25 samples; at N=50, the distribution peaks increased monotonically in accordance with the ground truth values; and for N=100, all distributions were clearly separated at sub-Hz precision levels.

From the perspective of conventional FFT analysis, these results are extraordinary: the standard inverse relationship between total temporal span and frequency precision suggests a resolution of ∼1 Hz should require ∼1 s of data, up to a constant of order unity. However, the Bayesian estimates here accurately separated 1 Hz frequencies with less than 20 ms of samples; in fact, data comprising just over half a cycle (e.g., 5 ms) gave standard deviations of 0.4 Hz or less in the retrieved posterior distributions. While surprising from the perspective of many traditional Fourier analysis techniques, this behavior is in fact well known and entirely consistent with previous analyses in Bayesian spectrum estimation, as first described by Jaynes [26] and extended by Bretthorst [36]. Intuitively, such improvements can be ascribed to the Bayesian model’s processing of noise that automatically suppresses fluctuations on the order of σ. Indeed, manual inspection of data such as those in Figure 4 certainly reveals clear differences between the curves that should be resolvable by curve fitting: Bayes’ theorem can reach these and similar intuitive conclusions mechanically, within a complete framework that incorporates all assumptions in a logically consistent fashion.

### 3.3. Anomaly Detection

The highly accurate, low-error results above provide strong validation of our Bayesian approach for parameter estimation from sensor data. Ultimately, though, the full probability distributions in Figure 5 furnish more detail than necessary for anomaly detection, which is interested in binary questions such as: Is the system operating as expected or not?

**Figure 6:**
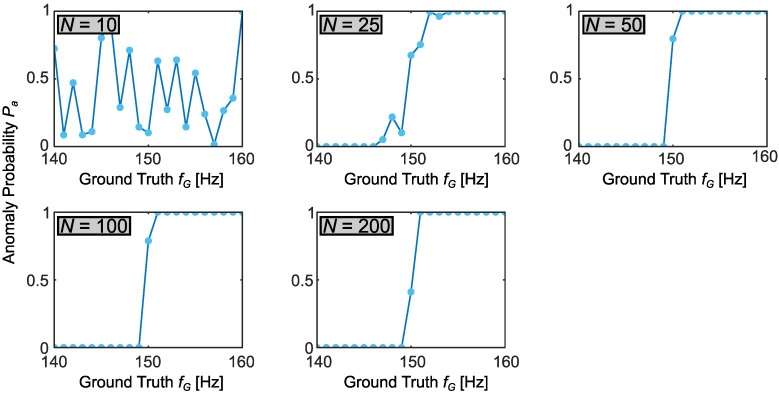
Anomaly detection curves for each sample number *N*. The anomaly probability $P_{a}$ is the Bayesian-inferred probability that the excitation frequency exceeds 150 Hz, plotted against the ground truth frequency.

As an example, suppose that the device corresponding to the sensor outputs in Figure 4 is designed for operation at frequencies below $f_{0}=\omega_{0}/2\pi =150$ Hz; any frequency above this entails an anomalous state. From the *R* frequency samples $\left\{\omega^{\left(r\right)}\right\}$ obtained in Bayesian inference, this anomaly probability $P_{a}=P\left(\omega >\omega_{0}\right)$ can be estimated as

(20) $$P_{a}\approx \frac{1}{R}\sum_{r=1}^{R}{𝟙}_{\left(\omega_{0},\infty \right)}\left(\omega^{\left(r\right)}\right),$$

i.e., the fraction of samples that exceed $\omega_{0}$. We computed $P_{a}$ for all 105 inference cases and plotted them in Figure 6 against ground truth frequency $f_{G}$, grouped according to number of time samples *N*. As a reference, a perfect detection curve with 100% accuracy and no uncertainty would be a step function with $P_{a}=0$ for all $f_{G}<150$ Hz and $P_{a}=1$ for $f_{G}>150$ Hz.

Unsurprisingly, given the full inference results above, N=10 time samples were insufficient to offer any meaningful estimate of an anomaly; this improved markedly at N=25 and was nearly ideal for N≥50. Indeed, if we define $P_{a}<0.1$ as high confidence that an anomaly has not occurred, and $P_{a}>0.9$ as high confidence that it has, the N=25 tests returned high-confidence results for all cases except $f_{G}\in \left\{148,149,150,151\right\}$, and for N≥50, the only ground truth frequency inside the transition region was $f_{G}=150$ Hz—the best performance possible under our test increments of 1 Hz.

To build these findings into a full anomaly detection scheme, one can define an anomaly threshold $T_{a}$ such that an alarm is sounded whenever $P_{a}>T_{a}$. With the null and alternative hypotheses associated with “normal” and “anomalous” operation, respectively, a type I error (false alarm) will thus occur whenever $P_{a}>T_{a}$ but $f_{G}<150$ Hz, whereas a type II error (missed detection) follows when $P_{a}<T_{a}$ but $f_{G}>150$ Hz [37]. Considering the N=25 case in Figure 6, for example, $T_{a}=0.1$ would admit type I errors for $f_{G}\in \left\{148,149\right\}$ Hz, but no type II errors for any $f_{G}$; by contrast, $T_{a}=0.9$ would avoid all type I errors, but experience a type II error at $f_{G}=151$ Hz. The *probabilities* for these errors in practice would depend on the specific attack—i.e., the distribution of frequencies an adversary selects compared to the distribution under normal operating conditions. If available, such attack knowledge could be incorporated into specifying a significantly more informative prior than the uniform one considered here, leading to even more efficient anomaly detection (in terms of fewer samples *N*) than suggested by the results of Figure 6 with $\pi_{0}\left(x\right)$ from Equation (8).

Regardless of the prior used, we expect the general tradeoff between accuracy and response time observed here to remain: with more samples *N*, the accuracy of steady-state parameter estimation increases steadily, yet so does the danger of missing a transient attack operating over a small number of samples only. In this regard, it would be interesting to explore the asymptotic behavior of our approach, perhaps using techniques such as those developed in the context of distributed denial-of-service attacks [38]. Nevertheless, because the limit N→∞ corresponds to an infinite record length, the asymptotic regime is insensitive to attacks of finite duration, and therefore faces vulnerabilities to transients. Accordingly, as we look toward applying our techniques in real-world systems, we suggest first performing numerical tests to determine the number of samples *N* required to achieve a detection curve of sufficient accuracy for any specific application. Then, inferences can be made on each successive length-*N* chunk, permitting a running update of the system’s state and thus facilitating responses to anomalies on time scales as short as the fundamental NΔt record length, which, as shown in our examples here, can be remarkably smaller than with non-Bayesian methods.

## 4. Discussion

Given the simplistic use case, which works well as a proof of concept for the use of our Bayesian method for anomaly detection, further investigations are warranted. The presented results could pave the way to future work for evaluating the robustness of the approach, e.g., comparisons to a basic FFT when there is a change in the conditions by introducing a slight nonlinearity in the model, a change in the noise distributions, or temporal correlations in the noise processes.

From a computational side, our use of pCN was motivated by its ease of implementation and our familiarity with it in previous work [31,32]. Yet it is possible that other MCMC methods could prove more efficient; in this application, potentially promising approaches include parallelized coupled chains [21,39], affine-invariant samplers [40], and posterior approximations [41]. Alternatively, one could draw on the analytical procedures outlined in the foundational work on Bayesian spectrum estimation, where, subject to reasonable approximations, nuisance parameters can be integrated out of the posterior distribution to leave a function of frequency only [26,36]. With such a one-dimensional posterior, direct numerical integration becomes a viable option, obviating the need for MCMC at all and making real-time estimation vastly more computationally efficient. Exploring the extent to which these simplifications can be applied to interesting cyber-physical problems will prove a crucial direction for future research.

## Figures and Tables

**Table 1:** Experimental actuator parameters.

Variable	Description
$E_{a}$	Armature voltage
$E_{b}$	Back EMF voltage
$R_{a}$	Armature resistance
$L_{a}$	Armature inductance
$J_{m}$	Rotational inertia
$B_{m}$	Viscous friction
$K_{T}$	Motor torque constant
$K_{E}$	Back EMF constant

## Data Availability

Data used in this study available from the authors upon reasonable request.
